# JRC GMO-Matrix: a web application to support Genetically Modified Organisms detection strategies

**DOI:** 10.1186/s12859-014-0417-8

**Published:** 2014-12-30

**Authors:** Alexandre Angers-Loustau, Mauro Petrillo, Laura Bonfini, Francesco Gatto, Sabrina Rosa, Alexandre Patak, Joachim Kreysa

**Affiliations:** Molecular Biology and Genomics Unit, Joint Research Centre, European Commission, Ispra, Italy

**Keywords:** Genetically Modified Organism, Matrix approach, Screening, qPCR

## Abstract

**Background:**

The polymerase chain reaction (PCR) is the current state of the art technique for DNA-based detection of Genetically Modified Organisms (GMOs). A typical control strategy starts by analyzing a sample for the presence of target sequences (GM-elements) known to be present in many GMOs. Positive findings from this “screening” are then confirmed with GM (event) specific test methods. A reliable knowledge of which GMOs are detected by combinations of GM-detection methods is thus crucial to minimize the verification efforts.

**Description:**

In this article, we describe a novel platform that links the information of two unique databases built and maintained by the European Union Reference Laboratory for Genetically Modified Food and Feed (EU-RL GMFF) at the Joint Research Centre (JRC) of the European Commission, one containing the sequence information of known GM-events and the other validated PCR-based detection and identification methods. The new platform compiles *in silico* determinations of the detection of a wide range of GMOs by the available detection methods using existing scripts that simulate PCR amplification and, when present, probe binding. The correctness of the information has been verified by comparing the *in silico* conclusions to experimental results for a subset of forty-nine GM events and six methods.

**Conclusions:**

The JRC GMO-Matrix is unique for its reliance on DNA sequence data and its flexibility in integrating novel GMOs and new detection methods. Users can mine the database using a set of web interfaces that thus provide a valuable support to GMO control laboratories in planning and evaluating their GMO screening strategies. The platform is accessible at http://gmo-crl.jrc.ec.europa.eu/jrcgmomatrix/.

## Background

Current legislations in the European Union (EU) foresee zero tolerance for unauthorised GMOs and stringent requirements for GMO approval and labelling [1-3]. Traceability is a key element in the implementation of EU Regulations, and relies on the availability of analytical methods for sensitive and accurate determination of GMO content. The European Union Reference Laboratory for Genetically Modified Food and Feed (EU-RL GMFF), hosted by the Joint Research Center (JRC) of the European Commission, must, pursuant to Article 32 of Regulation (EC) N. 882/2004, provide National Reference Laboratories with reference methods and tools for GMO analysis. Commission Regulation (EC) No 641/2004 requires the EU-RL GMFF to maintain a database containing GMO events sequence information.

The polymerase chain reaction (PCR) has proven to be the most accurate and reliable technique available for GMO detection, identification and quantification and is applicable to a wide range of samples, from seeds to highly processed food and feed. This technique is therefore widely used in GMO analysis. The PCR methods are generally grouped according to the specificity of the target sequence. The highest levels of specificity are achieved by event-specific methods that target the junction between the inserted DNA and the recipient genome, as this region is unique to each DNA integration event. Construct-specific methods target DNA sequences that span two different types of molecular entities, such as a promoter sequence and a gene sequence within a single transgenic construct. Element-specific methods target sequence elements commonly found in GMO constructs, such as the CaMV 35S promoter (P-35S) or the terminator of the *nopaline synthase* gene of *Agrobacterium tumefaciens* (T-nos). For the analysis of GMOs in the food and feed chains, a “screening” approach is generally followed by the GMO-control laboratories as an initial step in the analysis, in which a set of element- or construct-specific tests (also called screening methods), is performed to detect presence/absence of a range of GM events. Negative responses from a panel of such element- or construct-specific detection methods would eliminate the possibility of GMO presence in a test sample, assuming that the targets tested cover the GMO universe to be detected [4]. Positive signals can require the identification of the GMOs containing the detected GM-elements, and the confirmation of their presence with relevant event-specific methods. The preliminary identification of GMOs likely present in the sample can be achieved with the assistance of information matrices providing the expected signal patterns of known GMOs with a defined range of element- and construct-specific detection methods. These tools may narrow down the possible candidates or trigger un-authorized GMO alerts if none can be identified.

Recent efforts were made to build and distribute such information matrices, including the GMOseek [5] and GMOfinder [6] projects. These matrices integrate information from various sources, but mainly rely on laboratory testing, which is laborious and time-consuming, and on annotation information from publicly available sources, such as public versions of dossiers submitted by companies to regulatory authorities. This latter approach does not always provide reliable predictions of PCR-based detection as elements with the same name can vary at the level of their DNA sequences in different GMOs. Indeed, even if various public resources describe the annotations of known GM events (for example, see the Biosafety Clearing-House Living Modified Organism Registry, http://bch.cbd.int/database/lmo-registry/), the DNA sequence themselves are not always readily available.

To help bridging this gap, we present here a new information matrix called JRC GMO-Matrix that, unlike the others, is based on sequence data of known GM events as well as of primers and probe of methods that have been validated in a collaborative trial according to the principles and requirements of ISO 5725 and/or the IUPAC protocol [7]. This approach ensures a high reliability and consistency of the results provided. In addition, the JRC GMO-Matrix is built using bioinformatics tools and can be rapidly updated with additional methods or information on newly developed GMOs.

The JRC GMO-Matrix combines the information from two resources:

1. The GMOMETHODS database, the EU Database of Reference Methods for GMO Analysis [8] (http://gmo-crl.jrc.ec.europa.eu/gmomethods/). It supplies information on PCR assays validated according to international standards and is supplemented with methods that have been verified by the EU-RL GMFF for EU legal purposes. As of today (November 2014) the GMOMETHODS database contains 144 different DNA-based (PCR) methods of analysis for identifying 63 single GM events and 21 taxon-specific genes. It also provides screening assays for detection of different genetic elements, which have been used in the development of the majority of the GMOs approved anywhere in the world, today. In particular, the database contains 60 event-specific methods, 20 construct-specific methods, 26 element-specific methods and 38 taxon-specific methods. For each method the database provides core data required by analytical laboratories for implementing and carrying out the GM testing in line with the standards protocols. In particular, the GMOMETHODS database provides all primers and probes sequences and target genetic elements.
2. The Central Core DNA Sequences Information System (CCSIS), a molecular database that stores annotated GM event sequences either retrieved from public sequences databases or submitted to the EU-RL GMFF as part of the GMO authorization procedures [9]. The CCSIS currently stores more than 120 entries referred to DNA sequences of genetically engineered organisms, the majority of which cannot be made publicly available as they are considered Confidential Business Information (CBI) by the applicants.

To respect the necessity of confidentiality, in the JRC GMO-Matrix, the detection of each GMO-event by each method is pre-determined by *in silico* simulations of the PCR reaction (including probe binding if a probe is present). The results, but not the confidential sequence information, are stored in a local database that can be used, via a web interface, to build ad-hoc matrices, where the user can select the GMOs, the species, and the methods to prepare the final output. The JRC GMO-Matrix database is updated regularly to accommodate new detection methods and new GMO sequences that become available to the EU-RL GMFF.

## Construction and content

### JRC GMO-Matrix database generation

The information and scripts required to populate the database of the JRC GMO-Matrix application are installed and maintained in a restricted area of the JRC’s internal network. Binary versions of the tools “re-PCR” (developed by NCBI [10]) and “matcher” (from the EMBOSS package [11]) are installed locally on a high-performance computing platform, while the sequence information of the GM events and the detection methods are stored and automatically indexed in a separate server. The server hosting the JRC GMO-Matrix application contains and accesses only pre-computed values that are updated regularly. These pre-computed values consist of a “score” given to each pair of GMO/Detection Method, which can be “0” for no binding, “1” for imperfect primer/probe binding, and “2” for perfect primers and probe binding.

**Figure 1:**
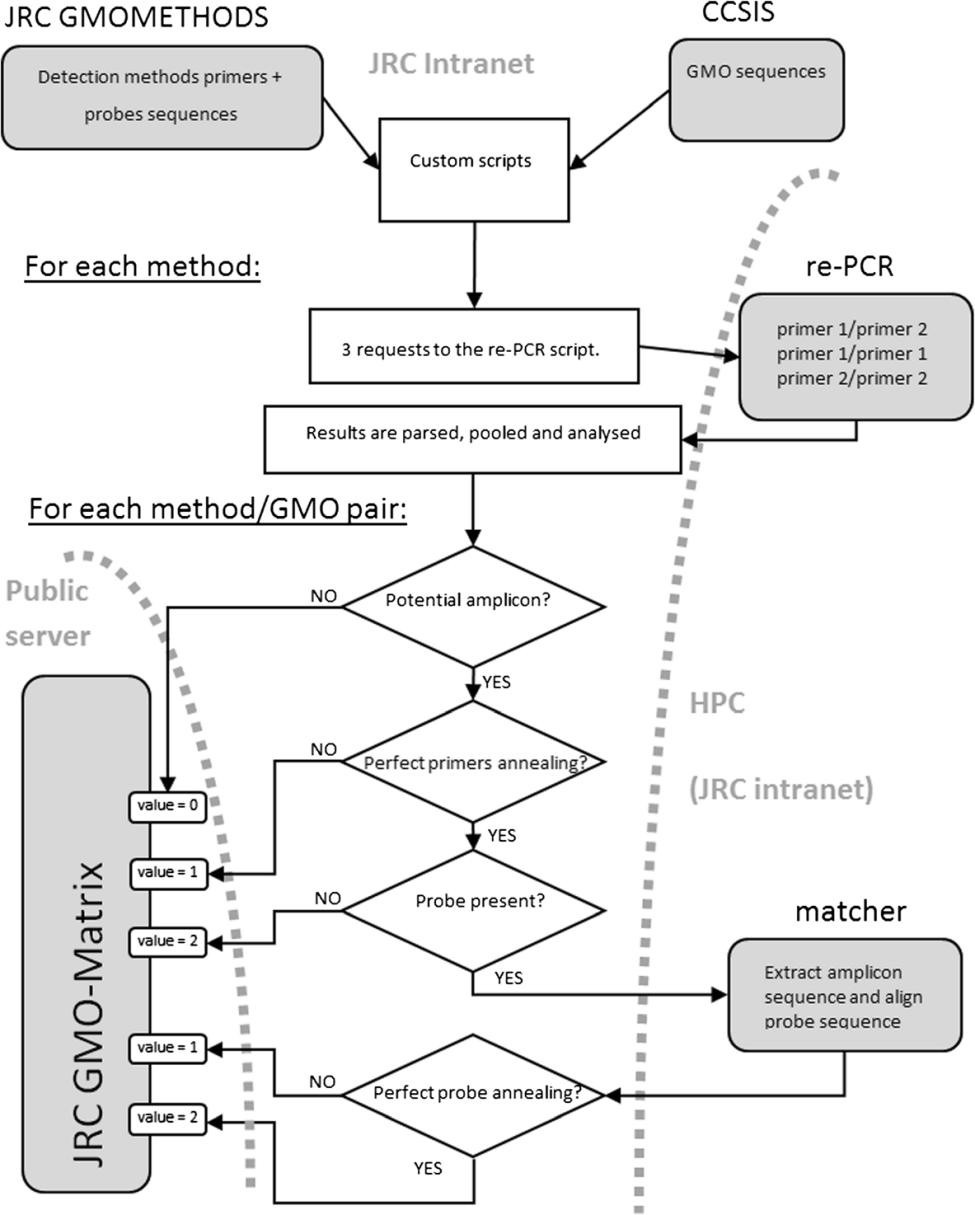
**Pipeline of the pre-calculations performed by the scripts in order to populate the database for the JRC GMO-Matrix application (Public server).** See Methods section for details.

This update is performed by a set of custom scripts (written in Ruby and PHP5) developed in-house that follows the pipeline shown in Figure 1:

1. For each detection method, three separate reverse electronic PCR (re-PCR) requests are performed, using all three possible pairs for the two primers (i.e. primer1 and primer2, primer1 and primer1, primer2 and primer2), with the following parameters: maximum gaps: 2, maximum mismatches: 2, amplicon length between 20 and 500 bp and a target database consisting of the properly indexed GMO sequences of the CCSIS.
2. The results are parsed to keep the identity of the GMOs identified by each re-PCR simulation and pooled for each detection method. For each hit, the number of gaps and mismatches are also saved, as well as, if the method has a probe, the beginning and ending base of the potential amplicon in the target sequence. The size of the amplicon generally varies between 50 and 200 bp, depending on the method. This latter information is then used, for each hit, to determine whether the probe sequence is complementary to the amplicon by extracting the amplicon sequence and aligning it to the probe using the application *matcher* of the EMBOSS package: the alignment tool is run twice (i.e. using the extracted amplicon and its reverse complement, as a probe can theoretically bind on each of the sequence strands) and the best produced alignment is taken into further consideration.
3. All the information is then transformed into a final value. For each detection method/GMO combination, the best hit is identified, and given a 2 value if it is a perfect match for both primers and probes, or a value of 1 for imperfect primer/probe match, which nevertheless might allow for binding and successful detection. If no hit is found for a GMO with the set thresholds of gaps and mismatches, the detection method/GMO combination is given a value 0.
4. The scripts export all these calculated values to a postgreSQL database set up for the JRC GMO-Matrix application, and used for building the responses to the requests.

The JRC GMO-Matrix application itself is a Ruby on Rails application (http://rubyonrails.org/) deployed on a server accessible to the outside of the JRC intranet, integrated with the EURL-GMFF public website (http://gmo-crl.jrc.ec.europa.eu/).

### Comparison of *in silico* simulations and experimental results

Experimental specificity tests were done on DNA solutions obtained or extracted from Certified reference materials (CRM) purchased from the Institute for Reference Materials and Measurements (IRMM, Geel, Belgium) of the European Commission or the American Oil Chemists’ Society (AOCS, Urbana, IL, USA).

DNA was extracted according to a modified CTAB methods (ISO21571, [12]) followed by additional purification on Genomic Tip 20 (Qiagen GmbH, Hilden, Germany) when necessary, or by using the Nucleospin® kit (Macherey-Nagel GmbH, Düren, Germany). DNA from cotton CRMs was extracted using the Foodproof GMO sample preparation kit (Biotecon Diagnostics, Potsdam, Germany) according to the manufacturer’s instructions

The DNA was quantified by fluorescence detection using PicoGreen® ds DNA quantitation kit (Life Technologies, Carlsbad, CA, USA) and was examined on agarose gel to verify its integrity. Inhibition runs [13] were performed for all samples to ensure that no PCR inhibitor was present in the extracts.

Each CRM was tested in duplicate in a 25 μL amplification mix containing 100 ng DNA, 2x TaqMan® Universal PCR Master Mix (Life Technologies, Carlsbad, CA, USA), 600 nM of each primer and 200 nM of the FAM/TAMRA-labelled probe and loaded onto a 96-well plate. The thermal profile used was: 50°C for 2 min, 95°C for 10 min, followed by 45 cycles of 95°C for 15 s and 60°C for 60 s. Data acquisition was set on the step at 60°C. RTi-PCR runs were performed using the 7900HT Fast Real-Time PCR System and the 7500 Real Time PCR System (Life Technologies, Carlsbad, CA, USA). The data were analysed using the SDS 2.4 and 7500 software v2.0.6, respectively.

## Utility and discussion

The JRC GMO-Matrix application uses the GMO sequence information stored in the CCSIS, that are received from companies as part of their EU legal obligations or extracted from public nucleotide and patent sequences databases [9], as well as the primers and probe sequences of the existing detection methods compiled in the GMOMETHODS database [8] to perform *in silico* determination of PCR amplification and, when applicable, probe binding using bioinformatics tools, such as *re-PCR* and *matcher*. The application contains all the element- and construct-specific, but also the event-specific detection methods, from the GMOMETHODS database.

### Database pre-calculations

As described in the *Construction and Content* section, the JRC GMO-Matrix application relies on a relational database that contains pre-computed values corresponding to the quality of matching between the methods primers and probe and each GMO sequence. Those pre-calculated values represent the results of *in silico* simulations of the detection of each GMO by each PCR-based detection method and form the core data used by the application for its different use cases. The pre-calculated values range from 0 (no amplification detected) to 2 (perfect annealing of both primers and probe). An intermediate score of “1” was chosen when a potential amplicon has been detected, despite imperfect binding of the primers and/or probe (up to an arbitrary threshold of maximum 2 gaps and 2 mismatches per primer). This latter case was included as comparisons of the scripts output with laboratory results have shown that it is difficult to predict the efficiency with which the detection method will detect the GMO, or if it will detect it at all, when a few mismatches or gaps are present in the primers or probe binding sites (data not shown).

### Experimental verification

In order to verify the correctness of the scripts calculations, a subset of 6 methods (five element-specific and one construct-specific) and 48 GM events were selected, and the determinations made by the JRC GMO-Matrix were compared with experimental results (Table 1). For the large majority of the cases (279/288), the simulations of the JRC GMO-Matrix corresponded to the experimental observations. The nine “discrepancies” were all pairs of events/methods with weak experimental positive signals but no *in silico* identified amplicon. Further investigations on these templates, using event-specific methods revealed traces of contaminations by other GMO(s) that could explain those weak signals (Table 1).

**Table 1:** **Comparisons between the results shown by the JRC GMO-Matrix (M) and observations made experimentally (E) for a subset of methods (columns) and events (rows)**

	**Methods**											
	**QL-CON-00-008 (CTP2-CP4EPSPS)**		**QT-ELE-00-004 (p35S)**		**QL-ELE-00-013 (tNos)**		**QL-ELE-00-014 (bar)**		**QL-ELE-00-016 (CryIAb/Ac)**		**QT-ELE-00-002 (pat)**	
**Events**	**M**	**E**	**M**	**E**	**M**	**E**	**M**	**E**	**M**	**E**	**M**	**E**
**ACS-GH001-3**	0	-	2	+	2	+	2	+	0	-	0	-
**MON-01445-2**	2	+	2	+	2	+	0	-	0	+	0	-
**MON-15985-7** ^*^	0	+	2	+	2	+	0	-	2	+	0	-
**MON-00531-6**	0	+	2	+	2	+	0	-	2	+	0	-
**DAS-24236-5** ^**^	0	-	0	+	0	-	0	-	0	-	2	+
**MON-88913-8**	2	+	2	+	0	-	0	-	0	+	0	-
**BCS-GH002-5**	0	-	0	-	0	-	0	-	0	-	0	-
**BCS-GH005-8**	0	-	1	+	2	+	2	+	0	-	0	-
**BCS-GH004-7**	0	-	2	+	2	+	2	+	0	-	0	-
**SYN-EV176-9**	0	-	2	+	0	-	2	+	0	-	0	-
**DAS-01507-1**	0	-	2	+	0	-	0	-	0	-	2	+
**SYN-E3272-5**	0	-	0	-	2	+	0	-	0	-	0	-
**DAS-59122-7**	0	-	2	+	0	-	0	-	0	-	2	+
**SYN-IR604-5**	0	-	0	-	2	+	0	-	0	-	0	-
**MON-00810-6**	0	-	2	+	0	-	0	-	0	-	0	-
**MON-00863-5**	0	-	2	+	2	+	0	-	0	-	0	-
**MON-88017-3**	2	+	2	+	2	+	0	-	0	-	0	-
**MON-00603-6**	2	+	2	+	2	+	0	-	0	-	0	-
**ACS-ZM003-2**	0	-	2	+	0	-	0	-	0	-	2	+
**MON-89034-3**	0	-	2	+	2	+	0	-	0	-	0	-
**SYN-BT011-1**	0	-	2	+	2	+	0	-	2	+	2	+
**MON-00021-9**	0	-	0	-	2	+	0	-	0	-	0	-
**DP-098140-6**	0	-	2	+	0	-	0	-	0	-	0	-
**SYN-IR162-4**	0	-	0	-	2	+	0	-	0	-	0	-
**MON-87460-4**	0	-	2	+	2	+	0	-	0	-	0	-
**DAS-40278-9**	0	-	0	-	0	-	0	-	0	-	0	-
**BPS-25271-9**	0	-	0	-	2	+	0	-	0	-	0	-
**MON-00073-7**	2	+	0	-	0	-	0	-	0	-	0	-
**ACS-BN005-8**	0	-	0	-	2	+	2	+	0	-	0	-
**ACS-BN003-6**	0	-	0	-	2	+	2	+	0	-	0	-
**ACS-BN008-2**	0	-	2	+	0	-	0	-	0	-	2	+
**ACS-BN007-1**	0	-	2	+	0	-	0	-	0	-	2	+
**ACS-BN001-4**	0	-	0	-	2	+	2	+	0	-	0	-
**ACS-BN004-7**	0	-	0	-	2	+	2	+	0	-	0	-
**ACS-BN002-5**	0	-	0	-	2	+	2	+	0	-	0	-
**ACS-OS002-5**	0	-	2	+	0	-	2	+	0	-	0	-
**ACS-GM005-3**	0	-	2	+	0	-	0	-	0	-	2	+
**MON-89788-1**	2	+	0	+	0	+	0	-	0	-	0	-
**DP-356043-5**	0	-	2	+	0	-	0	-	0	-	0	-
**DP-305423-1**	0	-	0	+	0	+	0	-	0	-	0	-
**ACS-GM006-4**	0	-	2	+	0	-	0	-	0	-	2	+
**BPS-CV127-9**	0	-	0	-	0	-	0	-	0	-	0	-
**MON-87701-2**	0	-	0	-	0	-	0	-	2	+	0	-
**MON-87705-6**	2	+	0	-	0	-	0	-	0	-	0	-
**MST-FG072-3**	0	-	0	-	2	+	0	-	0	-	0	-
**DAS-68416-4**	0	-	0	-	0	-	0	-	0	-	2	+
**MON-04032-6**	0	-	2	+	2	+	0	-	0	-	0	-
**KM-000H71-4**	2	+	0	-	0	-	0	-	0	-	0	-

The methods names refer to the GMOMETHODS database identification code, with in parenthesis the target sequences. Events names are the unique identifiers associated with the GM transformation events. In MON-01445-2 and in MON-88913-8 traces of the MON-00531-6 were detected. In MON-15985-7and MON-00531-6 traces of the event MON-01445-2 were detected. In DAS-24236-5 x DAS-21023-5, MON-89788-1 and DP-305423-1 traces of MON-04032-6 were detected.

-: no signal observed, +: positive signal observed. ^*^This event is a retransformation of event MON-00531-6, so both DNA sequences were used for the Matrix simulation. ^**^This event is only available as a stacked event (DAS-24236-5 x DAS-21023-5), which was used for the experimental tests.

Table 1 shows only one instance where, for these methods and events, a “1” score was predicted (i.e. amplification predicted, but with mismatches and/or gaps in the primer annealing sequence). Experimentally, this combination gave a clear positive results. However, other instances were found to produce results reported as weak positive or unclear responses (data not shown), showing the importance of empirical substantiation of these cases and the reason they were assigned an independent value in the matrix.

It should be noted that, in the re-PCR simulations, the scripts also test potential amplicons generated by the individual primers alone (i.e. forward primer with forward primer, reverse primer with reverse primer). Any amplicon detected in these cases would fall outside the “intent” of the method, and would thus most probably constitute an unexpected/unwanted detection signal. These specific cases are quite rare, but are highlighted in the matrices for warning purposes.

The pre-calculated set of values increases the efficiency of the JRC GMO-Matrix application, as it avoids performing a new computation for each new request. Moreover, such architecture ensures the protection of the confidential GMO sequence information stored in the CCSIS, in fact, all the scripts accessing and processing these sequences are confined to the Intranet of the JRC, and are not accessible from the outside. The JRC GMO-Matrix core data set is regularly updated as new information is added either to the CCSIS or the GMOMETHODS database.

In the first release version of the JRC GMO-Matrix application, the data can be interrogated by the users in two ways as described below, but other query options will be implemented based on the feedback received.

### The event/method matrix

**Figure 2:**
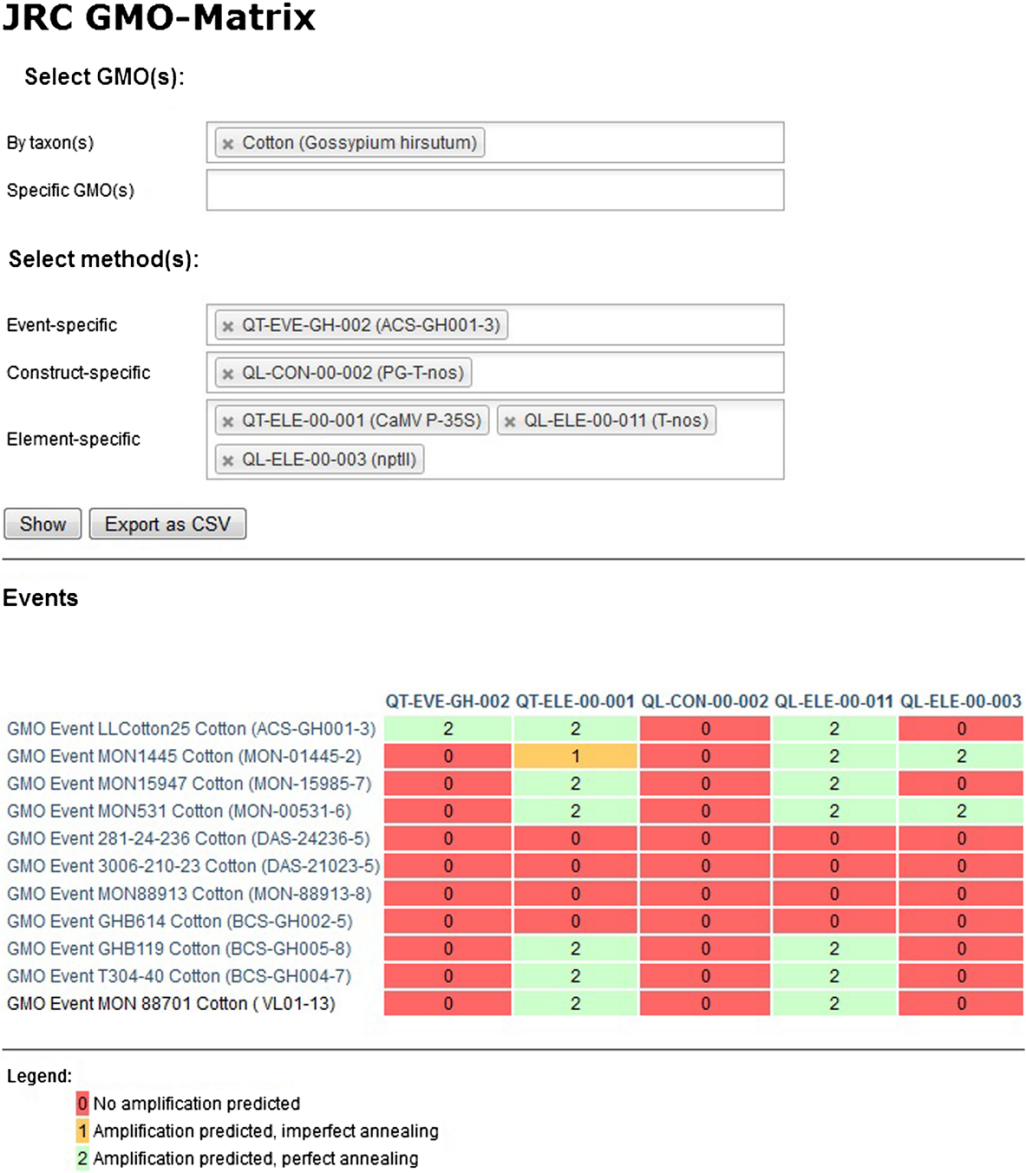
**Two-dimensional matrices (chosen GMO events in the Y axis versus chosen methods of the GMOMETHODS database on the X axis) built by the JRC GMO-Matrix application at the user’s request.**

The simplest use of the pre-computed values is to present them to the user, in the form of a two-dimensional matrix plotting, on one axis, selected detection methods and, on the other, selected GM events. The cells of the table then show the results of the *in silico* simulations (Figure 2).

The results can either be shown in the browser (as an HTML file) or exported as a Comma Separated Values (CSV) file that is compatible with spreadsheet applications. This information can visualize the general coverage of a set of selected detection methods for the GM events of a specific taxon, and allows identification of potential gaps. It can also be used to assess the specificity of new strategies involving sets of methods for their desired target GMO. In all instances, the JRC GMO-Matrix application simply fetches the values from its local database to populate the matrix, built on-the-fly and based on the user’s selection of methods and GMOs.

### GMO event finder

Alternatively, the pre-computed values can be used to identify potential GMOs present in a sample after experimental testing, based on the set of obtained positive and negative screening results.

**Figure 3:**
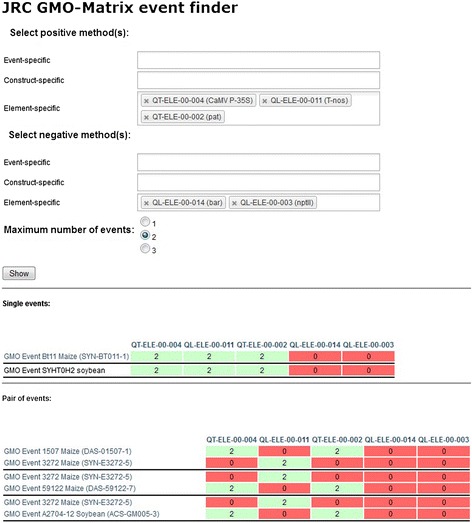
**GMO event finder interface of the JRC GMO-Matrix application.** The user selects the detection methods that were used experimentally to test the sample and selects the set of relative positive or negative results obtained. The application returns an array of GM events (or combinations thereof) that could match the observed results.

In this case, the user selects the screening method(s) that provided either a positive or negative result when a specific sample was analysed in the laboratory. The JRC GMO-matrix application then determines and shows which GM event(s) would fit the specified pattern, either individually or as mixes of up to 3 different events. The pre-computed values are, in this case, not immediately presented to the user, but used in a set of binary operations to determine which GM events (and combination thereof) fit the set conditions. A matrix similar to the one described in Database pre-calculations is then presented, which only includes those events (Figure 3).

This option assists the GMO control laboratories in the identification of the possible GMO(s) detected in the sample during this screening step, and narrows down the relevant event-specific methods to be chosen in the following analytical steps for identification and quantification.

### General considerations

As described in the introduction, the main difference between the information presented by the JRC GMO-Matrix and other existing GMO methods information matrices is the fact that our current tool involves *in silico* analyses at the level of the GMO events’ DNA sequences.

**Figure 4:**
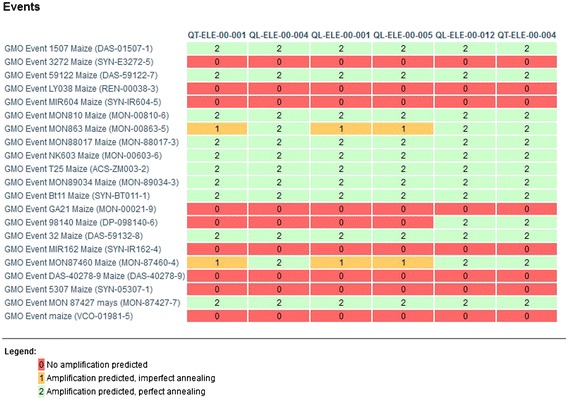
**GM event/method matrix highlighting inconsistent detection of the corn GM events by the six methods from the JRC GMOMETHODS database targeting P-35S, inconsistencies that can only be predicted by tools relying on sequence information such as the JRC GMO-Matrix application.**

Because of this, the JRC GMO-Matrix can correctly handle the fact that different elements representing the same GMO elements can have differences at the DNA sequence level in the different GMOs in which they are found. One clear example is the CaMV 35S promoter: as shown in Figure 4, a matrix containing the six CaMV 35S promoter detection methods in the GMOMETHODS database, shows patterns in the GM corn events that cannot be predicted by annotation alone, as in the case of DP-098140-6 maize event, which is not described to contain a CaMV 35S promoter in the transgenic cassette but is expected *in silico* to be detected by four of the six P-35S element-specific detection methods. This is explained by the presence of six CaMV 35S enhancer regions in the transgenic insert that have partially (but not completely) overlapping sequences with the CaMV promoter.

On the other hand, the requirement for complete DNA sequence information restricts the number of events that can be incorporated in the JRC GMO-Matrix application, in particular for new events or for events that have not been submitted for authorization in the European Union. As of November 2014, the application includes more than 80 GMO events, which comprehensively cover the GMOs authorized in the EU and represent a significant fraction of the GMOs reported in the Biosafety Clearing-House Living Modified Organism Registry, which currently lists less than 170 registered single events internationally. Building the missing sequence knowledge from publicly available sequence databases or from users contributions will continue to be an important future activity in the improvement of the CCSIS and the updates of JRC GMO-Matrix application.

The strict requirements that methods need to meet for selection and inclusion into the GMOMETHODS database ensure also high quality in terms of specificity and efficiency of the methods and reliability of the results provided by the JRC GMO-Matrix application (see [8]).

The EU-RL GMFF is supported in its functions by the ENGL (European Network of GMO control Laboratories) and has by this way access to a wealth of practical experience in GMO detection. This allows a constant monitoring of the information of the JRC-GMO matrix against laboratory data, which is an ongoing process that has already confirmed its consistency with laboratory results and will ensure that conflicts will be efficiently investigated.

## Conclusion

The correct implementation of legislations on GMO approval and labelling, both in the EU and outside, requires extensive testing of food and feed samples through the whole food chain. With the growing number and complexity of GMOs, the new tool presented here is expected to reduce the labour intensity and costs of testing for the presence or absence of GMOs in food and feed supply chains.

GMO-control laboratories currently perform an initial screening using a set of detection methods to identify the presence or absence of a range of elements commonly found in GMOs. Negative responses from such a panel of screening methods eliminate the possibility of GMO presence in a test sample, but only if the selected screening methods cover all the GMOs to be detected. In this frame, the JRC GMO-Matrix provides valuable support in selecting the validated screening methods for the optimal screening strategy.

In case of one or more positive signals, additional laboratory work is necessary to identify the actual GMO that produced the signals. The JRC GMO-Matrix also makes this step more efficient by displaying the list of GMO(s) consistent with the patterns of the results obtained from the screening. In case of an authorised GMO in the sample, the laboratory can then directly use an event-specific method for identification (verification of the identity) and quantification (for labelling purposes) of the detected GMO.

## Availability and requirements

The platform is deployed as a web interface, freely accessible, at http://gmo-crl.jrc.ec.europa.eu/jrcgmomatrix/
